# SPIN1 is a proto-oncogene and SPIN3 is a tumor suppressor in human seminoma

**DOI:** 10.18632/oncotarget.25977

**Published:** 2018-08-21

**Authors:** Damian Mikolaj Janecki, Marcin Sajek, Maciej Jerzy Smialek, Maciej Kotecki, Barbara Ginter-Matuszewska, Bogna Kuczynska, Anna Spik, Tomasz Kolanowski, Riko Kitazawa, Maciej Kurpisz, Jadwiga Jaruzelska

**Affiliations:** ^1^ Institute of Human Genetics, Polish Academy of Sciences, Poznan, Poland; ^2^ Department of Developmental, Molecular and Chemical Biology, Tufts University Medical School, Boston, Massachusetts, U.S.A.; ^3^ Institute of Pharmacology and Toxicology, Technische Universität Dresden, Germany; ^4^ Division of Molecular Pathology, Ehime University, Graduate School of Medicine, Shitsukawa, Toon City, Ehime, Japan

**Keywords:** human seminoma, PUM proteins, testis germ cell tumors, SPIN1, SPIN3

## Abstract

SPIN1 is necessary for normal meiotic progression in mammals. It is overexpressed in human ovarian cancers and some cancer cell lines. Here, we examined the functional significance and regulation of SPIN1 and SPIN3 in the TCam-2 human seminoma cell line. We found that while SPIN1 overexpression reduced apoptosis in these cells, SPIN3 overexpression induced it. Similarly, SPIN1 upregulated and SPIN3 downregulated CYCD1, which is a downstream target of the PI3K/AKT pathway and contributes to apoptosis resistance in cancer cell lines. It appears that SPIN1 is pro-oncogenic and SPIN3 acts as a tumor suppressor in TCam-2 cells. To our knowledge, this is the first report of SPIN3 tumor suppressor activity. However, both SPIN1 and SPIN3 stimulated cell cycle progression. In addition, using luciferase reporters carrying *SPIN1* or *SPIN3* mRNA 3′UTRs, we found that PUM1 and PUM2 targeted and repressed SPINs. We also found that PUM1 itself strongly stimulated apoptosis and moderately slowed cell cycle progression in TCam-2 cells, suggesting that PUM1, like SPIN3, is a tumor suppressor. Our findings suggest that acting, at least in part, through SPIN1 and SPIN3, PUM proteins contribute to a mechanism promoting normal human male germ cell apoptotic status and thus preventing cancer.

## INTRODUCTION

Apoptosis and cell cycle progression are crucial processes that regulate human germ cell numbers. These processes are critical for fertility, but also play pivotal roles in cancer [1, 2]. Elucidating the mechanisms behind these processes will improve our understanding of both infertility and germ cell tumors. Testis germ cell tumors (TGCT) often arise on the male infertility background [3]. Some genes that strongly influence germ cell apoptosis and cell cycle progression are posttranscriptionally regulated by PUM (pumilio) proteins [4, 5]. PUM proteins are well described, highly conserved factors that target mRNAs by binding short nucleotide consensus motifs (UGUANAUA; pumilio binding elements, or PBEs) located in the 3’ untranslated region (3′UTR) [6]. PBE recognition is mediated by a highly conserved PUF-domain in PUMs and depends on PUM protein cooperation with several cofactors [7]. The two PUM paralogues in mammals, *PUM1* and *PUM2*, are very similar in structure [8], and PUM1 is important for mammalian germ cell development [9].

*SPIN1* (also known as SPINDLIN1) was selected as a candidate mRNA target for PUM1 via a RIP-Chip screening of human HeLa cancer cells [10], as it binds PUM1 and contains several PBE-like motifs in its 3′UTR. *Spin1* was first identified as a maternal transcript specifically and abundantly expressed in unfertilized eggs and two-cell embryos in mice, fish, and pigs [11–13]. Cell cycle-dependent phosphorylation enables Spin1 to bind to the meiotic spindle [12]. Spin1 is necessary for meiotic resumption; Spin1-deficient mouse oocytes undergo normal folliculogenesis, but do not resume meiosis [14]. *Spin1* is largely homologous to Y-linked *Ssty* spermiogenesis-specific transcripts [15], including *Ssty1*, *Ssty2*, and many *Ssty-*like pseudogenes [16]. The Spin/Ssty gene family, including Spin1, is expressed during mouse and human male gametogenesis, but its function is still largely unknown [17].

SPIN1 was first associated with tumorigenesis in human ovarian cancer tissues [18]. Human SPIN1 overexpression increased the proportion of mouse NIH3T3 cells in S and G2/M phases, promoted proliferation and colony formation *in vitro*, and induced tumor formation in nude mice [19]. Similarly, high SPIN1 level was associated with greater malignancy in liposarcoma cells *in vitro*, and SPIN1 overexpression enhanced cell proliferation and reduced apoptosis through PI3K/AKT signaling [20]. SPIN1 depletion reduced proliferation and induced apoptosis [21]. However, SPIN1 overexpression in HeLa cells [22] and porcine oocytes [11] led to cell cycle arrest at metaphase, mitotic spindle dysfunction, multinucleation, and chromosome instability.

Here, we investigated the functional significance of SPIN1 and its structurally similar paralogue, SPIN3, with respect to apoptosis and cell cycle progression in the human seminoma cell line, TCam-2. This TGCT cell line represents human male germ cells at a very early stage of prenatal development [23]. TGCTs are the most common solid tumors in young men, and TGCT incidence is on the rise [24]. Because PUM1 may target *SPIN1* [10], we also assessed PUM1 and PUM2 regulation of SPIN1 and SPIN3, as well as the effects of PUM proteins on apoptosis in TCam-2 cells. Our results strongly suggest that SPIN1 is a proto-oncogene, while SPIN3 is a tumor suppressor.

## RESULTS

### SPIN1 downregulates and SPIN3 upregulates apoptosis in TCam-2 cells

**Figure 1:**
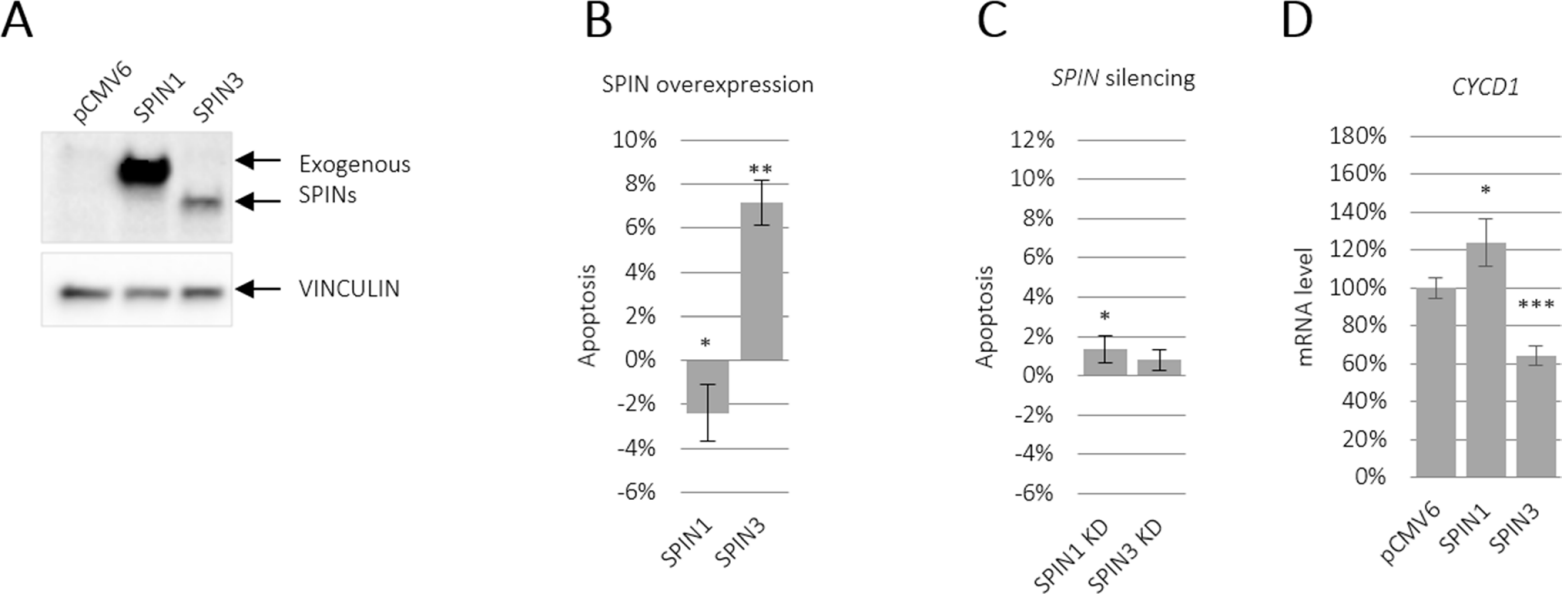
SPIN paralogues differentially influence TCam-2 cell apoptosis SPIN1 and SPIN3 were overexpressed or silenced in TCam-2 cells and apoptosis was assessed using flow cytometry. Representative western blot showing SPIN overexpression compared to VINCULIN (**A**). Apoptosis was analyzed in TCam-2 cells overexpressing SPINs (**B**) and in cells in which SPINs were silenced (**C**) CYCD1 expression was measured via real-time qPCR in cells overexpressing SPIN1 and SPIN3 (**D**). Cells transfected with an empty vector (overexpression) or control siRNA (knockdown) were the baselines in (B) and (C). ^*^P ≤ 0.05, ^**^P ≤ 0.005, ^***^P ≤ 0.0005.

SPIN1 downregulated apoptosis in liposarcoma cells [21]. To determine the effects of SPIN paralogues on apoptosis, we overexpressed SPIN1 and SPIN3 in TCam-2 cells and analyzed Annexin V staining via flow cytometry after 48 h. SPIN3 strongly increased and SPIN1 moderately decreased apoptosis (Figure 1B and Supplementary Figure 1). Importantly, SPIN3 overexpression was much lower than that of SPIN1 (Figure 1A). siRNA-mediated *SPIN1* knockdown increased apoptosis, although this effect was weak (Figure 1C and Supplementary Figure 2 left panel). Similarly, siRNA-mediated *SPIN3* knockdown weakly increased apoptosis, (Figure 1C and Supplementary Figure 2 right panel), likely due to much lower endogenous *SPIN3* levels compared to those of *SPIN1* in TCam-2 cells (Supplementary Figure 3). Because SPIN1 mediates PI3K/AKT signaling to promote apoptosis resistance in cancer cell lines [20], we performed real-time qRT-PCR to test whether SPIN1 or SPIN3 affected the downstream targets of that pathway. We assessed *CYCD1*, *AKT1*, *BCL2L1*, *NCL2L1*, *CREB1,* and *PIK3CA* mRNAs, and found that SPIN1 overexpression upregulated and SPIN3 overexpression downregulated *CYCD1* (Figure 1D and Supplementary Figure 4). The effects on *CYCD1* were in line with the anti-apoptotic effect of SPIN1 and pro-apoptotic effect of SPIN3.

### SPIN1 and SPIN3 promote TCam-2 cell cycle progression

**Figure 2:**
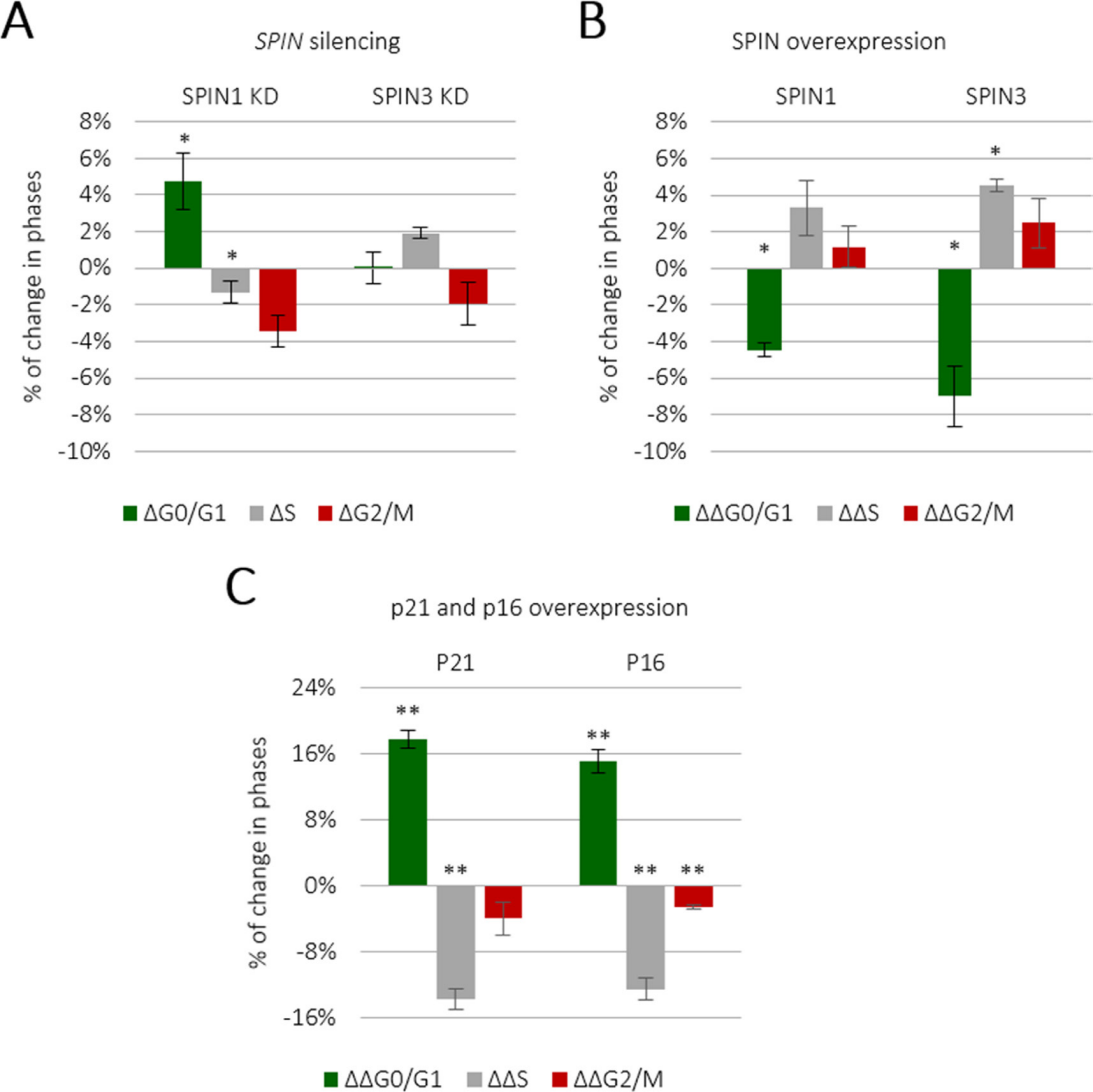
SPIN1 and SPIN3 promote TCam-2 cell cycle progression TCam-2 cell cycle analysis was performed following siRNA-mediated *SPIN1* or *SPIN3* knockdown (**A**) Cells transfected with control siRNA represented the baseline. ^*^P ≤ 0.05. TCam-2 cell cycle analysis was performed following *SPIN1* or *SPIN3* overexpression (**B**) Values higher than the baseline indicate an increase in a given cell population, while values below the baseline indicate a decrease. The p16 and p21 CDK inhibitors were used as positive controls (**C**).

Given that mouse Spin1 reportedly increased cell cycle rates [19], we sought to investigate whether human SPINs induced similar effects in TCam-2 cells. We knocked down individual *SPIN* genes using siRNA (Supplementary Figure 2) and analyzed the cell cycle via flow cytometry. *SPIN1* knockdown increased the population of cells in G0/G1 and decreased those in S and G2/M phases compared to controls (*P* ≤ 0.05) (Figure 2A and Supplementary Figure 5A). *SPIN3* knockdown had no significant effect (Figure 2A and Supplementary Figure 5A), possibly due to low endogenous levels as compared to *SPIN1* (Supplementary Figure 3). We then overexpressed SPIN1 and SPIN3 in TCam-2 cells and assessed cell cycle progression (Figure 2B and Supplementary Figure 5B), with p16 and p21 cyclin-dependent kinases (CDK), well known cell cycle inhibitors, as negative controls (Figure 2C and Supplementary Figure 5C) [25]. SPIN1 overexpression increased cell cycle progression, decreasing the number of cells in G0/G1 phase and increasing those in S and G2/M phases (Figure 2B and Supplementary Figure 5B). However, the effect of SPIN1 on TCam-2 cell cycling was weak as compared to previous reporting in NIH3T3 cells [19]. This could potentially be explained by the significantly longer TCam-2 cell doubling time (about 58 h [26]) compared to that of NIH3T3s (about 20 h) [19]. Moreover, Spin1 was stably overexpressed in NIH3T3s, while we employed transient overexpression and siRNA knockdown. SPIN3 had a moderately positive effect on cell cycle progression similar to that of SPIN1 (Figure 2B). p16 and p21 strongly inhibited TCam-2 cell cycle progression (Figure 2C and Supplementary Figure 5C).

In contrast to a previous report [22], we did not observe any morphological abnormalities, such as multinucleation, in TCam-2 cells following SPIN1 or SPIN3 overexpression 72 h post-transfection (Supplementary Figure 6). However, multinucleation was previously observed in HeLa cells stably overexpressing SPIN1 several days post-overexpression [22], and in porcine oocytes as soon as 12 h post-overexpression [11]. SPIN1-induced multinucleation may thus be dependent on specific cellular contexts.

### SPIN1 and SPIN3 mRNAs immunoprecipitate with PUM proteins

**Figure 3:**
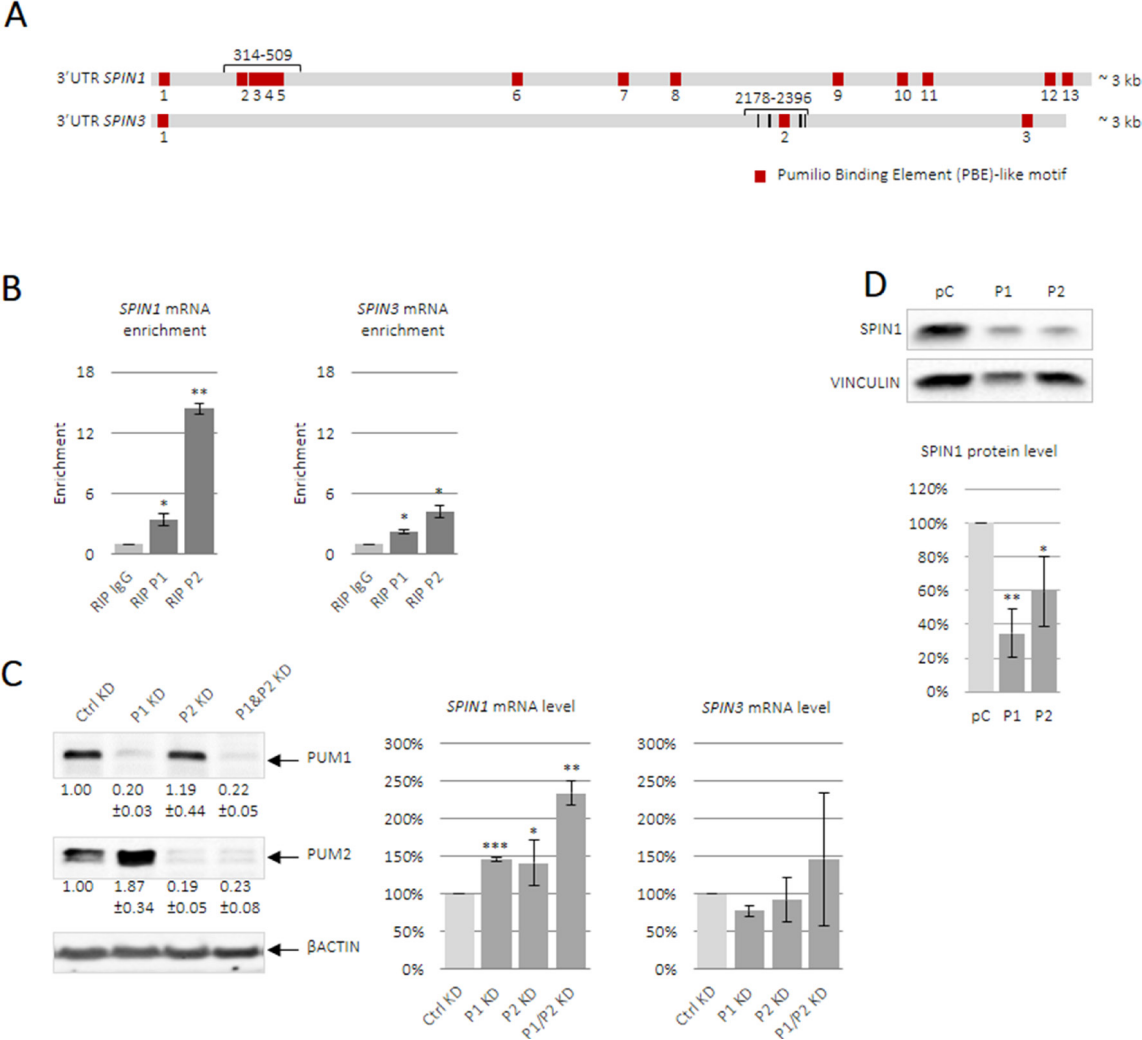
PUM1 and PUM2 proteins bind and regulate *SPIN1* and *SPIN3* mRNAs Schematic of full-length human *SPIN1* and *SPIN3* 3′UTRs (**A**). PBE-like motifs responsible for PUF-domain binding are in red and UGUA core motifs are in black (*SPIN3*). Short 3′UTR fragments containing PBE motifs, which were used for luciferase reporter assays, and full-length 3′UTRs are indicated in brackets, with position within the 3′UTR starting from the end of the stop codon. Enrichment of *SPIN1* and *SPIN3* in RIP-PUM1 and RIP-PUM2 (indicated by RIP P1 and RIP P2, respectively) was measured via RT-qPCR and was compared to the negative control (nonimmune IgG, indicated by RIP IgG) (**B**). Influence of PUM1 and PUM2 proteins on endogenous *SPIN* mRNA level (**C**). PUM1 and PUM2 siRNA knockdown efficiencies are shown on the left. Total RNA was isolated from TCam-2 cells in the presence of actinomycin D. *SPIN* expression was measured via RT-qPCR and was compared to that in untransfected cells. PUM1 or PUM2 overexpression downregulated endogenous SPIN1 as measured by western blotting (**D**). Graphs represent average values with standard errors. *P* ≤ 0.05, ^**^P ≤ 0.005, ^***^P ≤ 0.0005.

*Spin1* mRNA is reportedly posttranscriptionally regulated in two-cell mouse embryos [27]. Subsequently, human *SPIN1* was identified as a PUM1 target in HeLa cells [10]. PUMs recognize short UGUANAUA motifs located in mRNA 3′UTRs [7, 28]. After confirming that both *SPIN1* and *SPIN3* contained several PBE-like motifs (Figure 3A, Supplementary Table 1), we tested whether these bound PUMs in TCam-2 cells. We performed RNA immunoprecipitation (RIP) using specific anti-PUM1 and anti-PUM2 antibodies. The efficiencies of PUM1 and PUM2 binding to beads pre-coated with specific antibodies is shown in Supplementary Figure 7. Both anti-PUM1 and anti-PUM2 immunoprecipitates were enriched for *SPIN1* and *SPIN3* compared to the negative control (IgG) (Figure 3B).

### Effects of PUM1 and PUM2 on endogenous *SPIN1* and *SPIN3* mRNAs in TCam-2 cells

PUM interacts with the CCR4-NOT deadenylation complex [29]. When mRNA deadenylation is followed by storage [30], mRNA content does not change; however, when deadenylation is followed by degradation, repression by PUM entails decreased mRNA content. To determine the effects of PUM1 and PUM2 repression on endogenous *SPIN* mRNAs, siRNA-mediated *PUM1* and *PUM2* knockdown was performed in TCam-2 cells (Figure 3C). *PUM1* and *PUM2* knockdown upregulated *SPIN1* levels, and this effect was strongest under double *PUM1*/*PUM2* knockdown. *PUM1* and *PUM2* knockdown did not impact *SPIN3* mRNA levels. It is possible that PUM1- or PUM2-mediated repression leads to *SPIN1* mRNA degradation, but *SPIN3* mRNA storage [30].

### PUM1 and PUM2 overexpresion downregulates endogenous SPIN1 in TCam-2 cells

We overexpressed PUM1 and PUM2 in TCam-2 cells (Supplementary Figure 8) and measured SPIN1 protein levels via western blotting. SPIN1 was downregulated following PUM1 or PUM2 overexpression (Figure 3D). This experiment was performed in three biological replicates. This experiment was not performed for SPIN3, given its low endogenous levels in TCam-2 cells.

### PUM proteins downregulate expression of SPIN1 and SPIN3 luciferase reporters

**Figure 4:**
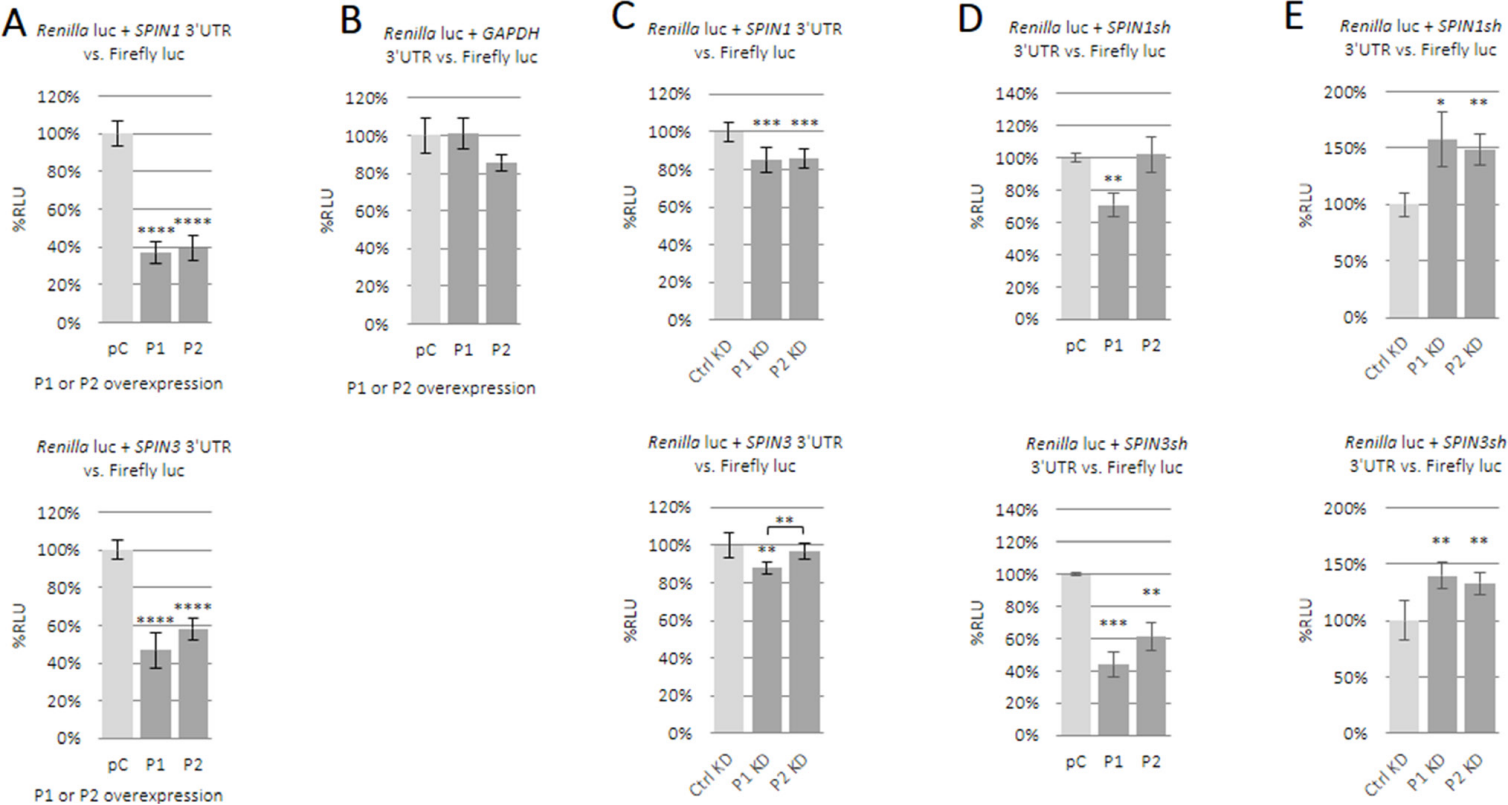
Influence of PUM1 and PUM2 proteins on luciferase reporter constructs carrying *SPIN1* or *SPIN3* 3′UTRs The effects of PUM proteins on *SPIN* expression were assessed using a dual luciferase assay. Luciferase reporter constructs carrying full-length 3′UTRs for *SPIN1* (upper panel) or *SPIN3* (lower panel) were tested with PUM1 (P1) or PUM2 (P2) overexpression or empty pCMV6-entry vector (pC) (**A**). Effects of PUM overexpression on luciferase reporter construct carrying full-length *GAPDH* mRNA 3′UTR, which lacks PBE motifs (negative control) (**B**). Effects of siRNA-mediated *PUM1* (P1 KD) or *PUM2* (P2 KD) knockdown (KD) on luciferase reporter constructs carrying full-length *SPIN* 3′UTRs (**C**). Effects of PUM1 or PUM2 overexpression (**D**). or knockdown (**E**). on luciferase constructs containing short *SPIN1* or *SPIN3* 3′UTR fragments. ^**^P ≤ 0.005, ^***^P ≤ 0.0005, ^****^P ≤ 0.00005.

Enrichment of *SPIN1* and *SPIN3* mRNAs in anti-PUM RIPs and SPIN1 downregulation by ectopic PUM1 or/and PUM2 encouraged us to investigate whether *SPIN* 3′UTRs are important for regulation by PUM proteins. Using luciferase reporters containing *SPIN1* or *SPIN3* full-length 3′UTRs in combination with PUM protein overexpression (Supplementary Figure 8A–8B), we found that both *SPIN*s were strongly repressed by PUM1 (Figure 4A upper panel) and PUM2 (Figure 4A lower panel). This effect was of nearly equal intensity for each PUM protein, indicating functional redundancy of PUM proteins. Expression of the luciferase reporter containing a full-length *GAPDH* mRNA 3′UTR, which does not contain any PBE-like motif, was unchanged (Figure 4B). To confirm *SPIN* mRNA regulation by PUM proteins, we performed siRNA knockdown of *PUM1* and *PUM2*. Despite efficient *PUM1* or *PUM2* knockdown (Figure 3C) we observed repression of the luciferase reporter constructs, although this repression was much weaker than that observed following PUM protein overexpression (Figure 4C). To understand this contradictory result, we assessed *SPIN1* and *SPIN3* 3′UTR fragments containing several PBE-like motifs, given that such motifs are reportedly crucial for PUM-mediated posttranscriptional regulation [7]. We prepared luciferase constructs containing very short *SPIN1* and *SPIN3* 3′UTRs fragments (195 nt and 218 nt in length, respectively), each containing several PBE-like motifs (four in the *SPIN1* short 3′UTR; one PBE-like motif and two motifs containing the UGUA core in the *SPIN3* short 3′UTR). PUM overexpression repressed these reporter constructs, with the exception of PUM2 and the SPIN1 construct (Figure 4D). PUM1 and PUM2 knockdown led to derepression of each reporter (Figure 4E). These results confirm that the *SPIN1* and *SPIN3* 3′UTR fragments containing PBE-like motifs are important for PUM-mediated repression.

### PUM1 upregulates apoptosis in TCam-2 cells

**Figure 5:**
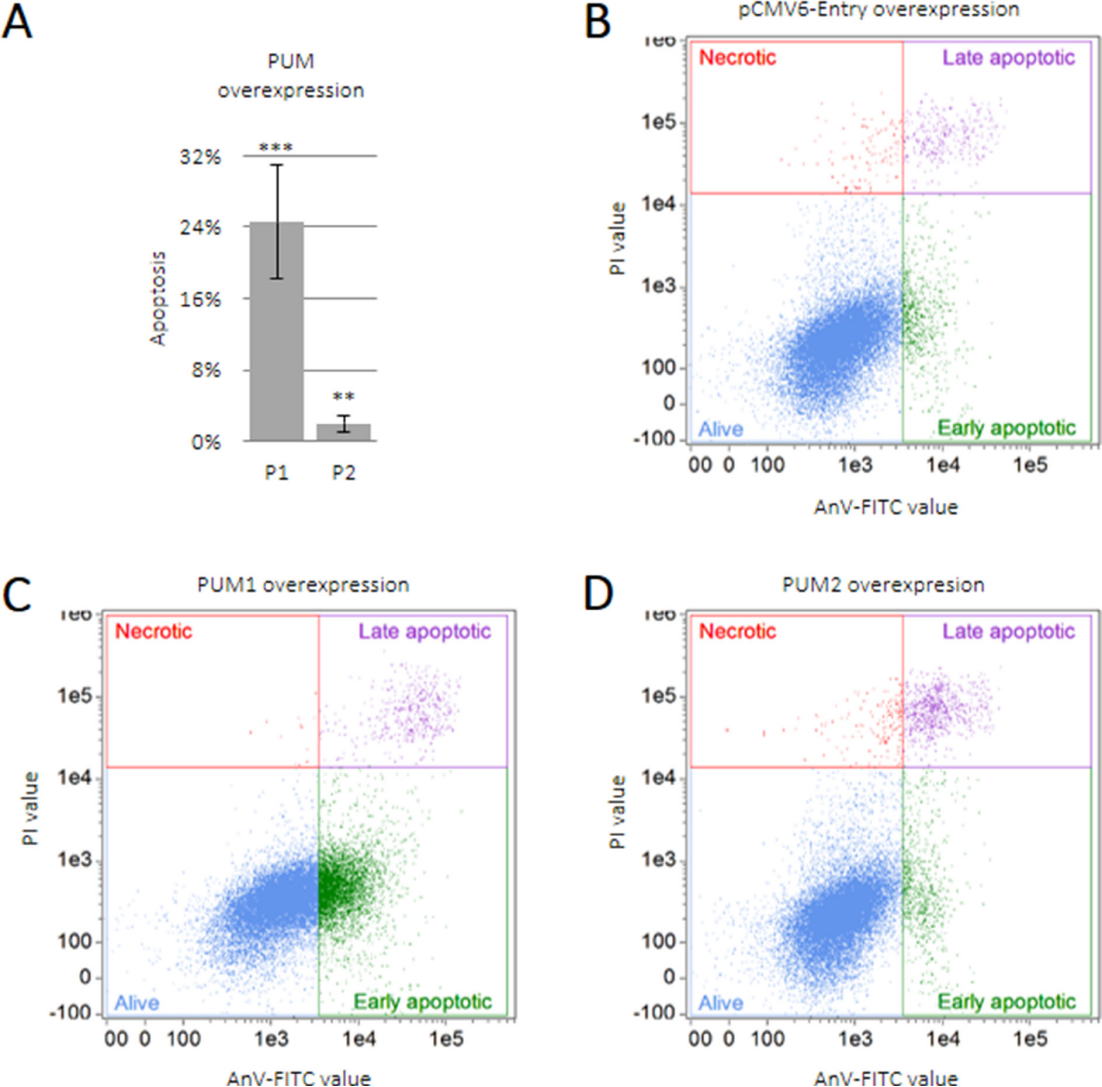
PUM1 induces TCam-2 cell apoptosis TCam-2 cells were transfected with constructs encoding PUM1 (P1), PUM2 (P2), or empty vector and cultured for 48 h. Apoptosis was measured as described for SPINs (**A**). Dot-plot showing quality of TCam-2 cell separation into living, necrotic, and early or late apoptotic populations after transfection with empty vector (**B**), PUM1 (**C**), or PUM2 (**D**) constructs.

We found that PUM proteins regulate the anti-apoptotic and pro-apoptotic effects of SPIN1 and SPIN3, respectively, in TCam-2 cells (Figure 1). To assess the effects of PUM proteins on apoptosis, we overexpressed PUM1 and PUM2 in TCam-2 cells. PUM1 strongly induced apoptosis (25% of cells underwent apoptosis) whereas PUM2 only weakly induced apoptosis (2% of cells underwent apoptosis) (Figure 5).

### PUM1 and PUM2 downregulate TCam-2 cell cycling

**Figure 6:**
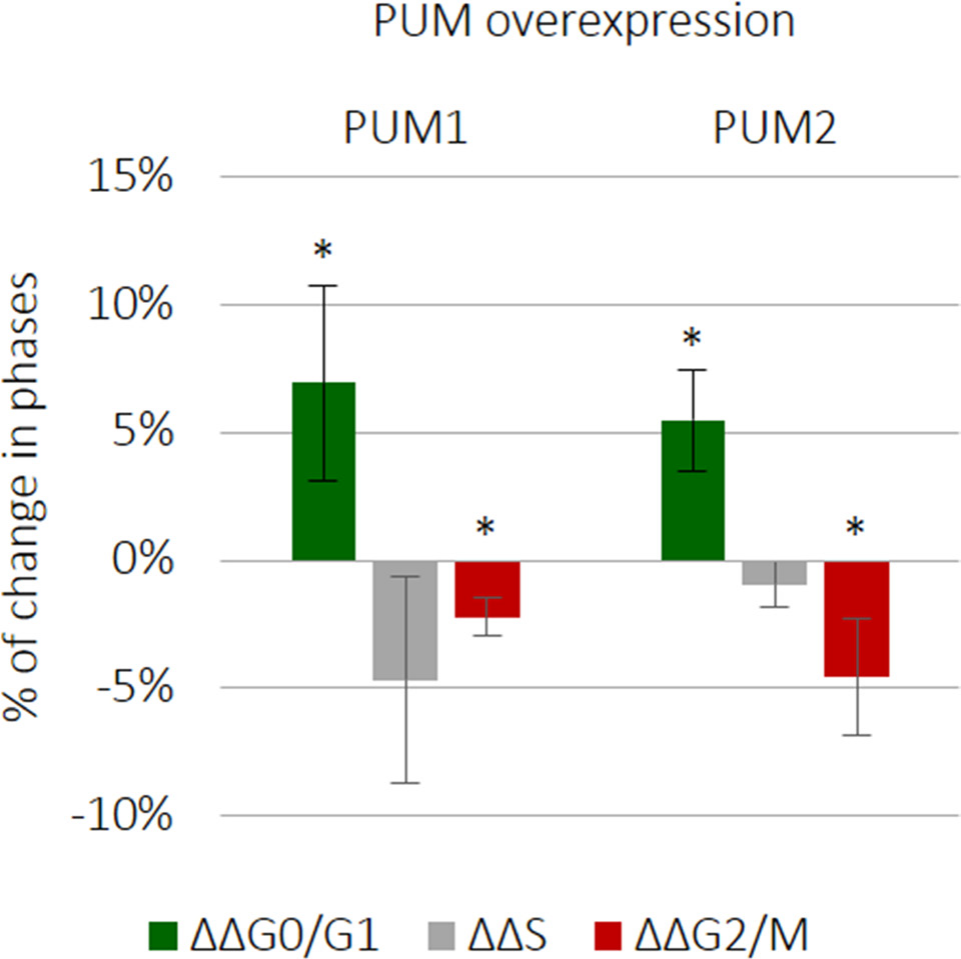
PUM1 and PUM2 slightly downregulate TCam-2 cell cycle progression PUM1 and PUM2 were separately overexpressed and the effects on cell cycle progression were measured 72 h later.

We found that SPIN1 and SPIN3 influenced TCam-2 cell cycle progression (Figure 2). To test whether PUM proteins influence TCam-2 cell cycling, we overexpressed PUM1 and PUM2. Both PUMs reduced cell cycle progression (Figure 6 and Supplementary Figure 9), increasing the proportion of cells in G0/G1 phase (7% for PUM1 and 5% for PUM2), and decreasing those in G2/M phase. These PUM-mediated effects on cell cycling, although significant (*P* ≤ 0.05) (Supplementary Figure 9), were not as prominent as the effects of PUM1 on apoptosis (Figure 5) (induction from 0–25% of cells, *P* ≤ 0.0005).

### SPIN3 overexpression is downregulated in TCam-2 and HeLa cells, but not in HEK293T cells

**Figure 7:**
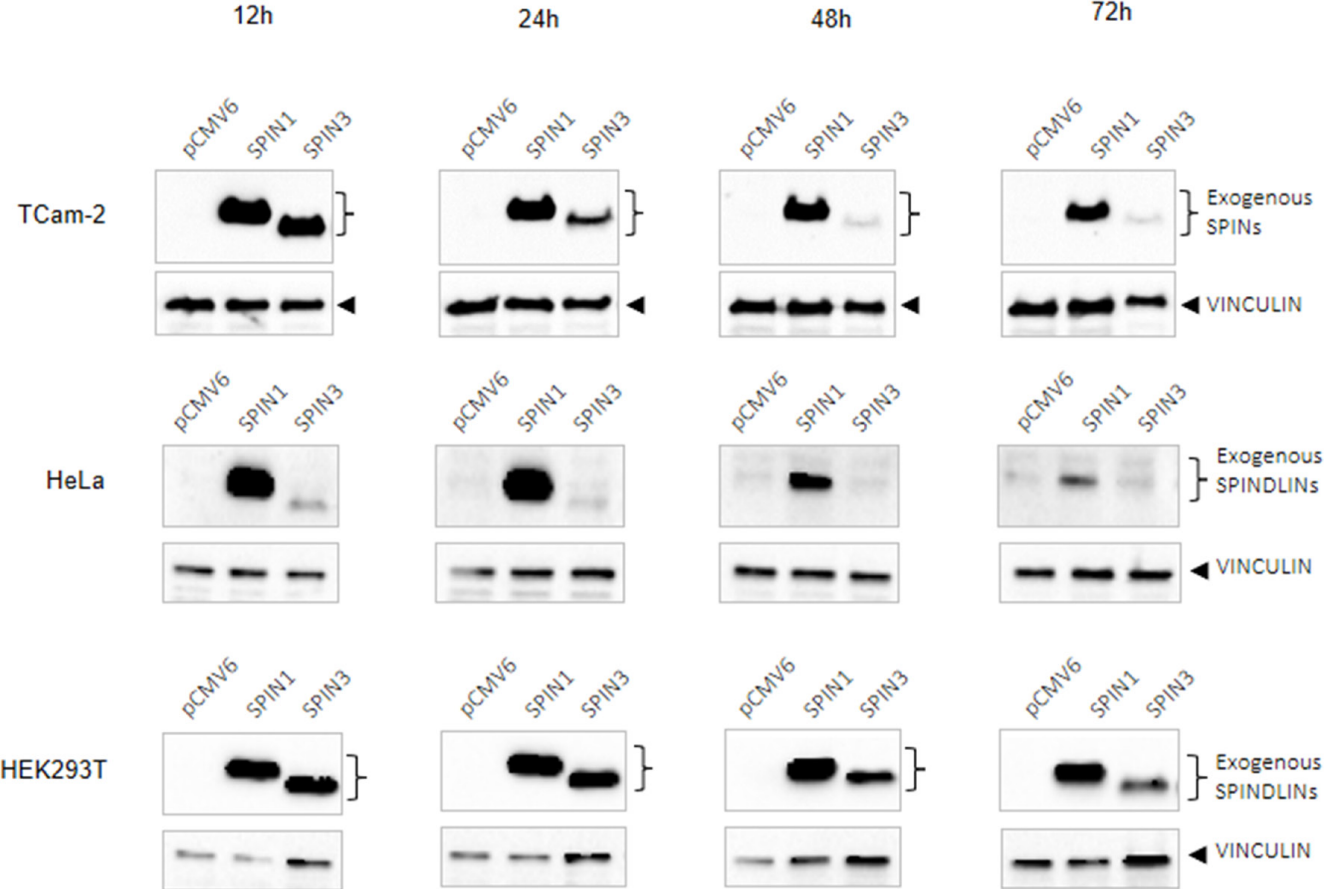
Western blots showing SPIN protein overexpression time-course SPIN overexpression in TCam-2 (upper panels), HeLa (middle panels), and HEK293T cells (lower panels) at 12, 24, 48 and 72 h of culture2. VINCULIN was used as loading control.

We observed that SPIN1 downregulated and SPIN3 upregulated apoptosis (Figure 1B). Given that TCam-2 male germ cells originate from a seminoma, we hypothesized that SPIN1 is a proto-oncogene and SPIN3 is a tumor suppressor in these cells. We found that in TCam-2 transfected cells SPIN3 overexpression was strongly downregulated (24 h of culture) compared to that of SPIN1 (Figure 7 upper panel). To test whether this pattern was TCam-2 cell specific, we overexpressed SPIN proteins in HeLa cells, which originate from tumor tissues, and in HEK293T cells, which do not. SPIN3 was strongly downregulated in HeLa cells (12 h of culture) as compared to SPIN1 (Figure 7 middle panel), but was not downregulated in HEK293T cells (Figure 7 lower panel). SPIN3 downregulation may thus be typical only for tumor-derived cells, but not for non-tumor cells, supporting our hypothesis that SPIN3 might be a tumor suppressor.

## DISCUSSION

Apoptosis is crucial in both normal germ cell development and in cancer. Apoptosis is fine-tuned and controls germ cell numbers in mammals, helping to ensure fertility. In tumors, however, apoptosis is downregulated, promoting unchecked tumor cell proliferation. The present study describes two proteins, SPIN1 and SPIN3, that play opposite roles in regulating apoptosis in the human seminoma (a type of TGCT) cell line, TCam-2. We showed that SPIN1 downregulates and SPIN3 upregulates apoptosis in these cells. We also demonstrated that the effects of these two proteins on apoptosis are at least partially due to their influence on CYCD1 expression. CYCD1 is a downstream target of the PI3K/AKT pathway that promotes apoptosis resistance in cancer. In agreement with previous findings in glioma cells [20, 31], we found that SPIN1 overexpression upregulated *CYCD1*. SPIN3, which has not yet been described, and which we found to be pro-apoptotic, downregulated *CYCD1*. Consequently, SPIN1 appears to play a pro-oncogenic role in TCam-2 cells, while SPIN3 is a tumor suppressor. SPIN1 also stimulated cell cycle progression in our study, as was previously reported in other types of cancers [18–21, 31]. In contrast with its proposed role as a tumor suppressor, SPIN3 also promoted cell cycle progression, and this requires further study.

Neither SPIN1 nor SPIN3 influenced cell proliferation (data not shown). According to previous reports, the effects of SPIN1 on cell proliferation may depend on cellular context. While SPIN1 promoted HeLa [32] and liposarcoma [21] cell proliferation, it did not have any demonstrable impact on breast cancer cell proliferation [31].

To the best of our knowledge, mechanisms controlling SPIN expression have not been heavily explored. *SPIN1* regulation might be 3′UTR-dependent in mice at the oocyte-embryo transition [27] and in cancer cells involving miR-489 [20, 31]. We predicted that PUM proteins could mediate *SPIN* posttranscriptional regulation, given the presence of several PBE-like motifs in the *SPIN1* and *SPIN3* mRNA 3′UTRs. We also observed that PUM1 or PUM2 overexpression downregulated endogenous SPIN1. *SPIN1* and *SPIN3* mRNAs were enriched in anti-PUM1 and anti-PUM2 immunoprecipitates, suggesting their direct interaction. This was confirmed by the downregulation of luciferase reporters carrying complete *SPIN1* or *SPIN3* mRNA 3′UTRs when coexpressed with PUM1 or PUM2. Unexpectedly, however, *PUM* gene knockdown weakly, but significantly, repressed luciferase reporters carrying *SPIN* 3′UTRs. To better understand this phenomenon, we generated luciferase reporters carrying either short *SPIN1* 3′UTR fragments or one *SPIN3* PBE-like motif and four UGUA motifs. PUM overexpression repressed these short 3′UTR reporters (except PUM2 overexpression in the case of *SPIN1*), while PUM silencing led to derepression in all cases. This indicates that PUM indeed targeted and repressed the regions containing PBE-like motifs within these 3′UTRs. The unexpected repression of full-length 3′UTR reporters following PUM1 or PUM2 silencing might indicate an additional pathway involving other RNA-binding proteins that may be acting in parallel to PUM repression and independent of PBE motifs.

Interestingly, PUM2 did not repress the reporter construct carrying the short *SPIN1* 3′UTR fragments. It is possible that for SPIN1 repression, PUM2 requires an additional site in the *SPIN1* 3′UTR that was missing in the short fragments, and this may reflect functional differences between PUM1 and PUM2. However, PUM2 overexpression was significantly lower than that of PUM1, which could also explain this result.

We observed different fates for repressed *SPIN1* vs. *SPIN3* mRNA targets. This might reflect 3′UTR-dependent recruitment of different cofactors for assembly of effector complexes leading to target mRNA storage vs. degradation. We also found that PUM1 itself strongly stimulated apoptosis and moderately slowed cell cycle progression in TCam-2 cells. These characteristics suggest that PUM1 is a tumor suppressor, a feature that has not yet been reported for PUM proteins in mammalian models. Given that PUM proteins regulate many mRNA targets [10], *SPIN1* is very likely one of numerous PUM1-repressed targets with similar impacts on apoptosis in TCam-2 cells.

Although SPIN1 and SPIN3 share highly similar amino acid sequences, we observed prominent functional differences between these two proteins. The most conserved central and C-terminal regions encompass three highly conserved functional Tudor domains that specifically recognize H3K4 methylation [33, 34]. Binding to methylated lysine is required for SPIN1 to promote proliferation and reduce apoptosis in liposarcoma cells [21]. Therefore, the N-terminal regions, which are much more divergent than the Tudor domain regions, may underlie the functional differences between SPINs, and could mediate interactions with different cofactors specific to each SPIN. Recent global interactome datasets from HEK293T cells show that indeed, SPIN1 and SPIN3 likely interact with different sets of proteins [35]. SPIN1, but not SPIN3, was shown to interact with TOPORS and PAX3 proteins, which are both involved in regulating proliferation and apoptosis. SPIN3, but not SPIN1, was shown to interact with NUDT1 and PFDN4, which are involved in DNA repair and protein folding, respectively [35].

Notably, SPIN3 overexpression in our experiments was markedly weaker and was lost much more quickly over time than that of SPIN1. This exogenous expression imbalance between pro-oncogenic SPIN1 and tumor-suppressing SPIN3 may reflect a mechanism used by TCam-2 cells to maintain tumor phenotype. In line with this, we observed similar patterns in HeLa cervical carcinoma cells overexpressing SPIN1 and SPIN3, but not in HEK293T cells, which do not originate from a tumor.

*SPIN* gene duplication during evolution likely gave rise to functional diversification, with individual paralogues playing different and even opposite roles in regulating TGCT and male germ cell development. In conclusion, our results suggest that at least in part through SPIN1 and SPIN3, PUM proteins may work as part of a mechanism promoting normal germ cell apoptotic status and thus preventing cancer.

## MATERIALS AND METHODS

### Cell culture and transfections

TCam-2 cells (supplied by our collaborator Dr Kitazawa) were cultured in RPMI with GlutaMAX medium (Life Technologies, Poland) supplemented with 10% (v/v) fetal bovine serum (FBS; HyClone, USA) and 1% (v/v) antibiotic/antimycotic solution (Lonza, Switzerland). Cells were transfected with plasmids or siRNA using the Neon Transfection System (Life Technologies), according to the manufacturer’s protocol, followed by culture in the same medium without antibiotic/antimycotic solution.

### Nuclei morphology analysis

TCam-2 cell nucleus localization and morphology was observed 72 h post-transfection, followed by 1 h incubation in culture medium supplemented with 1 ug/ml Hoechst 33258 (BD, USA). Cells were washed with 1× PBS (Lonza), and then visualized using a Leica DMi8 IVD microscope under UV (for Hoechst 33258 detection) and visible light.

### siRNA knockdown

For transient knockdowns, TCam-2 cells were transfected with one of the following specific siRNAs at 10 nM final concentration: *SPIN1* (sc-92696), *SPIN3* (sc-91032), *PUM1* (sc-62912), *PUM2* (sc-44773) or control (sc-37007) (Santa Cruz Biotechnology, USA). Knockdown efficiency was measured via reverse transcription and quantitative PCR (RT-qPCR) and/or western blot 72 h post-transfection. To measure *SPIN* mRNA levels following *PUM1* or *PUM2* knockdown, cells were incubated 6 h before lysis in culture medium supplemented with 5 μg/ml actinomycin D (Sigma-Aldrich, Germany) to cease transcription.

### Reverse transcription and quantitative PCR

To measure *SPINs*, *PUMs, CYCD1*, *AKT1*, *BCL2*, *BCL2L1*, *CREB1,* and *PIK3CA* mRNA levels, total RNA from cell cultures was isolated using TRIzol^®^ Reagent (Life Technologies) according to the manufacturer’s protocol. RNA was treated with DNase I (Sigma Aldrich) and reverse-transcribed using the Maxima First Strand cDNA Synthesis Kit (Life Technologies) according to the manufacturer’s protocol. Total cDNA was used as a template for qPCR amplification. The reaction was carried out using the CFX96 Touch™ Real-Time PCR Detection System (Bio-Rad, Poland) in 20 µl volumes containing 10 mM Tris-HCl, pH 8.3, 50 mM KCl, 3.5 mM MgCl_2_, 0.2× Sybr Green, 0.2 mM dNTPmix (dATP, dCTP, dGTP, dTTP), 0.2 µM F and R primers, and 0.5 U JumpStart™ Taq DNA Polymerase (Sigma Aldrich). Specific primers are listed in Supplementary Table 2. Amplification parameters were as follows: initial denaturation 95° C, 2.5 min and 40 cycles of: denaturation 95° C, 15 sec, annealing 10 sec (annealing temperatures for each primer pair are given in Supplementary Table 2), extension 72° C, 15 sec. β-ACTIN and *GAPDH* were used for normalization.

### Western blot analysis

Cells were lysed directly on plates via scraping in 2× Laemmli Sample Buffer (Bio-Rad). Protein lysates were subjected to SDS-PAGE followed by western blotting under standard conditions using nitrocellulose membranes and horseradish peroxidase (HRP)-conjugated secondary antibodies. Chemiluminescence was detected using the Clarity™ Western ECL Substrate (Bio-Rad) and developed in the ChemiDoc™ XRS+ system (Bio-Rad). Protein levels were analyzed semi-quantitatively using ImageLab 5.1 software (Bio-Rad). Signal intensities were normalized to their appropriate loading controls, β-ACTIN or VINCULIN.

### RNA immunoprecipitation

RNA immunoprecipitation (RIP) was performed using the Magna RIP™ RNA-Binding Protein Immunoprecipitation Kit (Merck Millipore, Germany) according to the manufacturer’s protocol. For one reaction, 10 µg antibody or IgG fraction from non-immune goat serum was used per 100 μl of Magnetic Beads. RNA for reverse transcription was isolated from immunoprecipitates using the RNeasy^®^ Plus Micro Kit (Qiagen, Germany). The PUM protein-magnetic bead binding efficiency was tested via western blot.

### Antibodies

This study used the following primary antibodies: anti-DDK (OriGene Technologies, TA50011, USA) for detection of proteins in the pCMV6-entry vector system 1:2500, anti-β-ACTIN (Sigma Aldrich, A2066) 1:10000, anti-SPIN1 (Abcam, Ab118784, UK) 1:1000, anti-PUM1 (Abcam, Ab3717) 1:5000, anti-PUM2 (Santa Cruz Biotechnology, sc-31535) 1:500. Secondary antibodies included: donkey anti-goat IgG-HRP (Santa Cruz Biotechnology, sc-2020) 1:50000, goat anti-rabbit IgG-HRP (Sigma Aldrich, A6154) 1:25000, goat anti-mouse IgG-HRP (Santa Cruz Biotechnology, sc-2005) 1:10000.

### Apoptosis analysis

Detection of apoptotic TCam-2 cells was performed 48 h post-transfection using the Annexin V-FITC Apoptosis Detection Kit (Beckman Coulter, USA) according to the manufacturer’s protocol, followed by flow cytometry using the FlowSight apparatus (Amnis). Results were analyzed using Image Data Exploration and Analysis Software (IDEAS^®^; Amnis).

### Cell cycle analysis

For cell cycle analysis following SPIN or PUM overexpression, 2 × 10^6^ cells were transfected with 30 μg of plasmid DNA encoding SPINs, PUMs, or an empty plasmid in the pCMV6-entry vector system (OriGene Technologies), plus GFP-F in the pEGFP-F vector as a marker of transfected cells (GFP-positive cells) in a 5:1 (plasmid DNA:pEGFP-F vector) ratio. Constructs encoding p21 and p16 or an empty plasmid in the pcDNA3 vector system with GFP-F co-transfection were used as positive controls. Transient knockdown was performed under standard conditions as described above. After transfection, cells were cultured in 15-cm plates for 72 h in standard medium. TCam-2 cells were subsequently trypsinized, washed with PBS, and fixed in cold 100% methanol on ice for 10 min. Cells were incubated at 37°C for 15 min in 50 µg/ml propidium iodide (PI; Sigma Aldrich) containing 330 µg/ml RNAseA (Sigma Aldrich), incubated 1 h on ice, and then analyzed using an S3e™ Cell Sorter (Bio-Rad). Data files were analyzed using ModFit LT software (Verity Software House).

### Luciferase assays

For luciferase assays, 150,000 cells were transfected with 1.5 μg of plasmid DNA encoding PUM1, PUM2, or an empty plasmid in the pCMV6-entry vector system (OriGene Technologies), plus the full-length or short 3′UTR of *SPIN1* or *SPIN3* in the psiCheck2 dual luciferase vector system (Promega, Germany) in a 10:1 (plasmid DNA:luciferase vector) ratio. Transfected cells were cultured in 12-well plates for 24 h in standard medium. For *PUM* transient knockdown experiments, 100,000 cells were transfected with 10 nM siRNA and 150 ng of psiCheck2 vector constructs as described above, and then cultured in 12-well plates for 48 h to achieve effective *PUM* mRNA depletion. Transfections were performed in three technical repeats per experiment. Cells were lysed and luminescence was measured two times using a Glomax-Multi Detection System luminometer (Promega) and the Dual-Luciferase Reporter Assay (Promega) according to the manufacturer’s protocol. Average *Renilla* to firefly luciferase luminescence ratios and standard deviations were calculated from three experiments. Luminescence ratios for each combination of constructs and/or siRNA were presented as % relative luciferase units (RLU). The sample transfected with empty pCMV6-entry or control siRNA plus the reporter construct was considered as 100%.

### Constructs

Constructs encoding PUM1 (RC201219), PUM2 (RC211307), SPIN1 (RC201938), or SPIN3 (RC215063), in the pCMV6-entry vector system for protein overexpression were purchased from OriGene Technologies. For luciferase assays, the full-length *GAPDH* (201 nt) *SPIN1* (3429 nt), or *SPIN3* (3339 nt) 3′UTRs or their fragments, SPIN1 (195 nt) and SPIN3 (218 nt), were cloned into the psiCheck2 vector (Promega) with the *Renilla* luciferase ORF, using NotI and XhoI restriction sites.

### Accession numbers

mRNA accession numbers were as follows: *GAPDH* NM_002046.5, *SPIN1* NM_006717.2, *SPIN3* NM_001010862.2, *PUM1* NM_014676 and *PUM2* NM_015317.

### Statistical analysis

Unpaired Student’s *t*-test was used to compare two groups. *P* < 0.05 was considered a statistically significant difference.

## SUPPLEMENTARY MATERIALS FIGURES AND TABLES


