# Supplementary material for: SPIN1 is a proto-oncogene and SPIN3 is a tumor suppressor in human seminoma
Source: Oncotarget. 2018 Aug 21;9(65):32466–77. doi: 10.18632/oncotarget.25977 (PMC6126697; doi:10.18632/oncotarget.25977)
Supplement: Supplementary file 1 [file oncotarget-09-32466-s001.pdf]

SPIN1 is a proto-oncogene and SPIN3 is a tumor suppressor in human seminoma

SUPPLEMENTARY MATERIALS

Supplementary Table 1: PBE-like motifs within the 3’UTRs of different SPINDLIN genes

No.	Sequence 5′→3′ [12 nt]	Position in mRNA [in 3′UTR]
1.	UUUGUAGACAUA	1152 [48]
2.	GAUGUAUGUAUC	1483 [379]
3.	UCUGUAUCUACC	1493 [389]
4.	UGUGUAUACAUA	1517 [414]
5.	AGUGUACACACA	1552 [449]
6.	GGUGUAAGUAAU	2441 [1337]
7.	CAUGUAAACACA	2827 [1723]
8.	CAUGUAAAUCUC	3018 [1914]
9.	UAUGUAUAAAAU	3612 [2508]
10.	CCUGUAUAUUUG	3847 [2743]
11.	GUUGUAAAUGUC	3939 [2835]
12.	UGUGUAGACAGU	4386 [3282]
13.	UCUGUAAGUAAU	4445 [3341]

[SPIN1 mRNA–4535 nt, 3′UTR – 3430 nt].

No.	Sequence 5′→3′ [12 nt]	Position in mRNA [in 3’UTR]
1.	UCUGUAGACACA	1159 [40]
2.	AUUGUAAAUUAG	3433 [2314]
3.	CAUGUAUUUACA	4314 [3195]

[SPIN3 mRNA–4479 nt, 3′UTR – 3359 nt].
None contained the classical UGUANAUA motif. We selected only those containing the UGUA core and two additional nucleotides from the AUA 3′ trinucleotide.

Supplementary Table 2: Primers used for real-time qPCR

Name	Sequence 5′→3′	Primer length [nt]	Amplicon length [bp]	Annealing temperature
β-ACT_F	CACCACACCTTCTACAATG	19	162	60° C
β-ACT_R	TAGCACAGCCTGGATAG	17		
ARNT_F	CCACAG-GAACTCTTAGGAA	19	117	54° C
ARNT_R	CATGACAGACAGCACTTG	18		
GAPDH_F	CGGAGTCAACGGATTTGGTCGTAT	24	307	56° C
GAPDH_R	AGCCTTCTCCATGGTGGTGAAGAC	24		
PUM1_F	TGATGTGATGGACAAGAC	18	97	56° C
PUM1_ R	AGAGTTCTGGCAATTACC	18		
PUM2 _F	CAGCCACAGTCACTACAG	18	78	60° C
PUM2_ R	GCAGCCATAAGGATGAGTT	19		
SPIN1_F	TACCAACTCTTAGATGAT	18	177	53° C
SPIN1_R	TTCTACTTGATGAATGAC	18		
SPIN3_F	ATGAACCTCTAACACAGT	18	158	53° C
SPIN3_R	AACTCTATTAGGAAGGACTT	20		
AKT1_F	TATTGTGAAGGAGGGTTG	18	80	60° C
AKT1_R	CATTCTTGAGGAGGAAGT	18		
CREB1_F	GAGTCAGTGGATAGTGTAA	19	98	58° C
CREB1_R	GCATCAGAAGATAAGTCATT	20		
PIK3CA_F	GTGGATGTGATGAATACTT	19	162	56° C
PIK3CA_R	TGGAATAAGATGGCATTG	18		
BCL2_F	GATGACTGAGTACCTGAA	18	113	58° C
BCL2_R	AGGAGAAATCAAACAGAG	18		
CYCD1_F	GGAGGAGAACAAACAGAT	18	85	56° C
CYCD1_R	CGGATTGGAAATGAACTT	18		
BCL2L1_F	CTTACCTGAATGACCACCTA	20	80	56° C
BCL2L1_R	ATTGTTCCCATAGAGTTCCA	20		

Primers are shown for *PUM1*, *PUM2*, *SPIN1*, and *SPIN3* siRNA-mediated silencing efficiency, and for *SPINs*, *CYCD1*, *AKT1*, *BCL2*, *BCL2L1*, *CREB1* and *PIK3CA* mRNA measurements in TCam-2 cells.

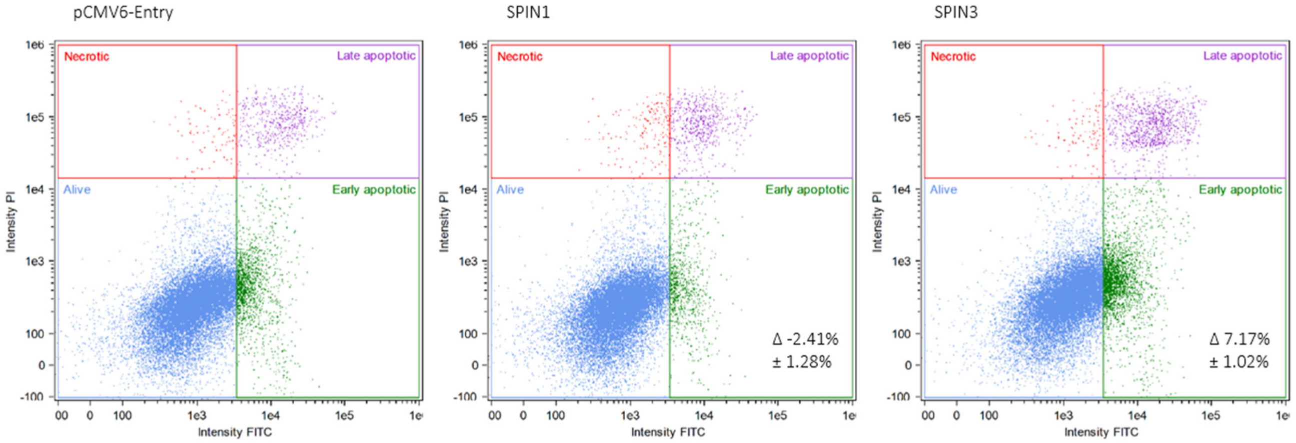

Supplementary Figure 1: Apoptosis in TCam-2 cells overexpressing SPIN1 or SPIN3. Dot-plots represent the quality of TCam-2 cell separation into to living, necrotic, and early or late apoptotic populations following SPIN1 or SPIN3 overexpression as compared to the negative control (TCam-2 cells transfected with empty pCMV6-entry vector).

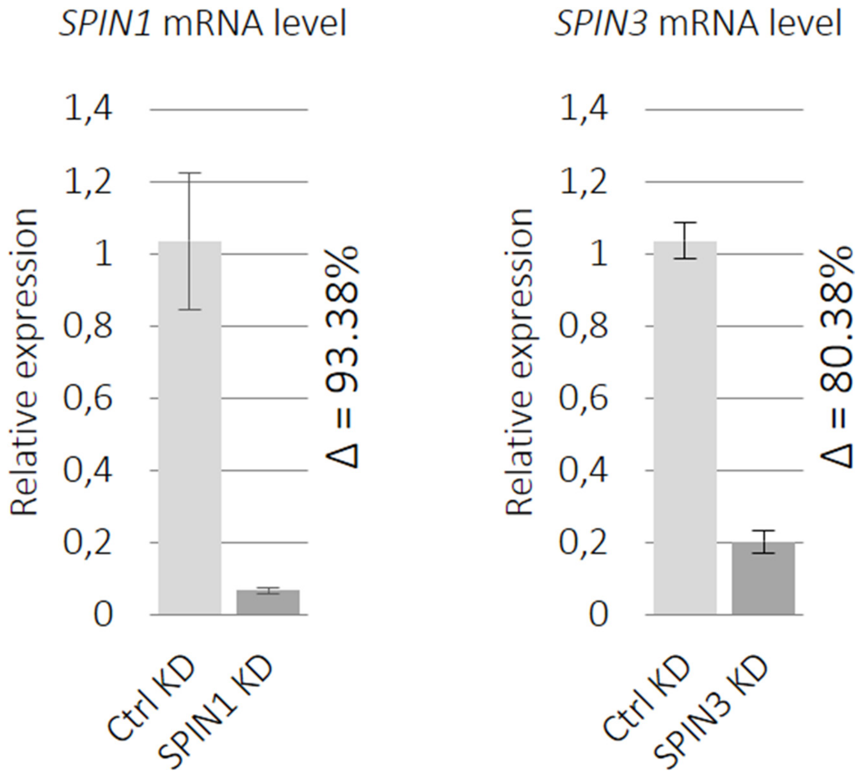

Supplementary Figure 2: Efficiencies of siRNA-mediated *SPIN1* or *SPIN3* knockdown as measured via RT-qPCR. *β-ACTIN* and *GAPDH* were used for normalization.

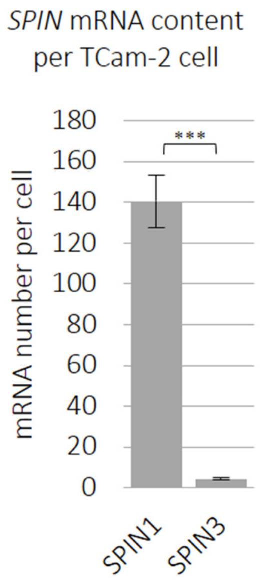

	SPIN1	SPIN3
Avarage value	140,47	4,50
Standard deviation	12,89	0,55

Supplementary Figure 3: Endogenous *SPIN1* and *SPIN3* mRNA levels in TCam-2 cells as measured via RT-qPCR.

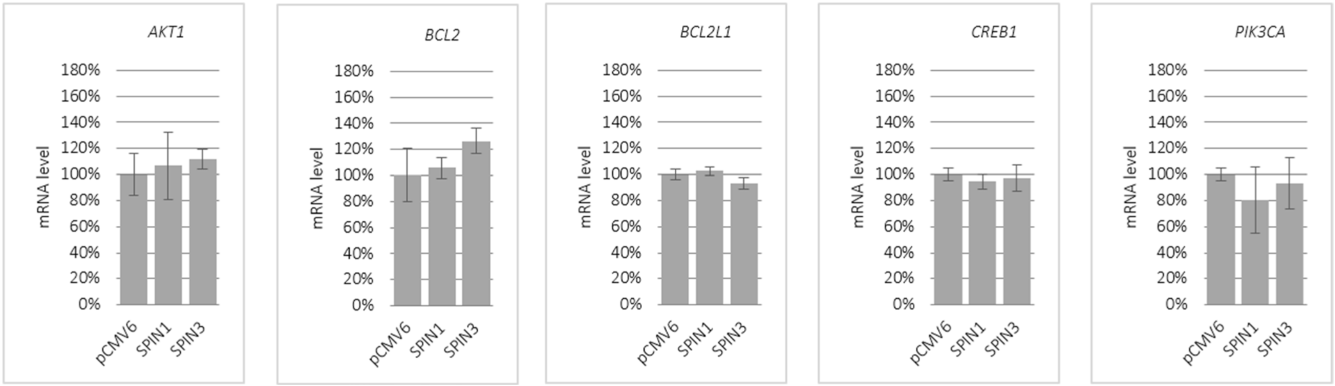

Supplementary Figure 4: Real-Time PCR measurements of AKT1, BCL2, BCL2L1, CREB1 and PIK3CA. To assess involvement of SPINs in PI3K/AKT pathway signaling, *AKT1*, *BCL2*, *BCL2L1*, *CREB1,* and *PIK3CA* levels were measured via real-time qPCR in cells overexpressing SPIN1 or SPIN3.

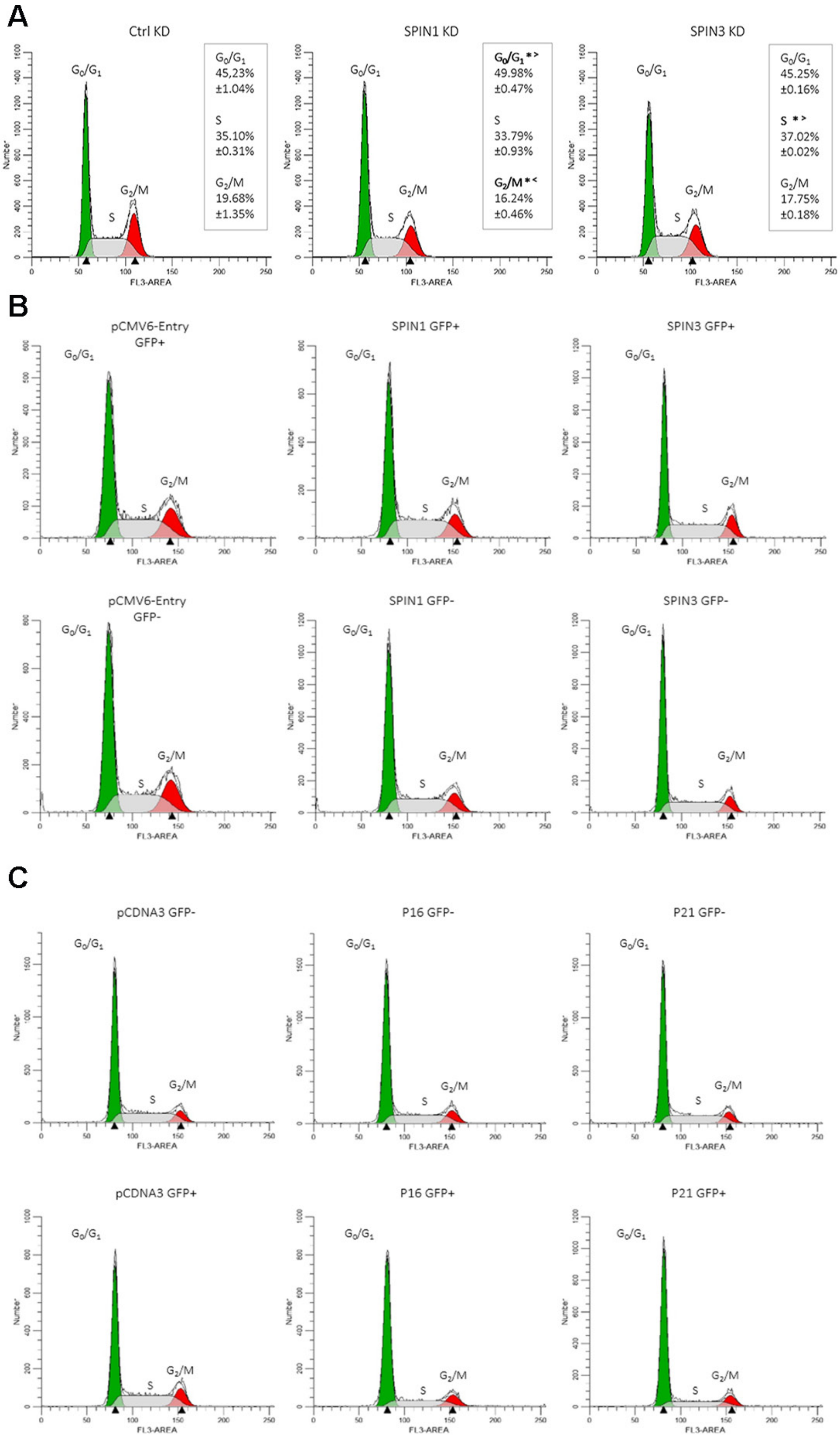

Supplementary Figure 5: Graphs showing separation quality and numbers of TCam-2 cells in different cell cycle phases as generated by ModFit LT software (Verity Software House). siRNA-mediated *SPIN* knockdown (**A)** SPIN overexpression. (**B)** p16 and p21 overexpression (**C).**

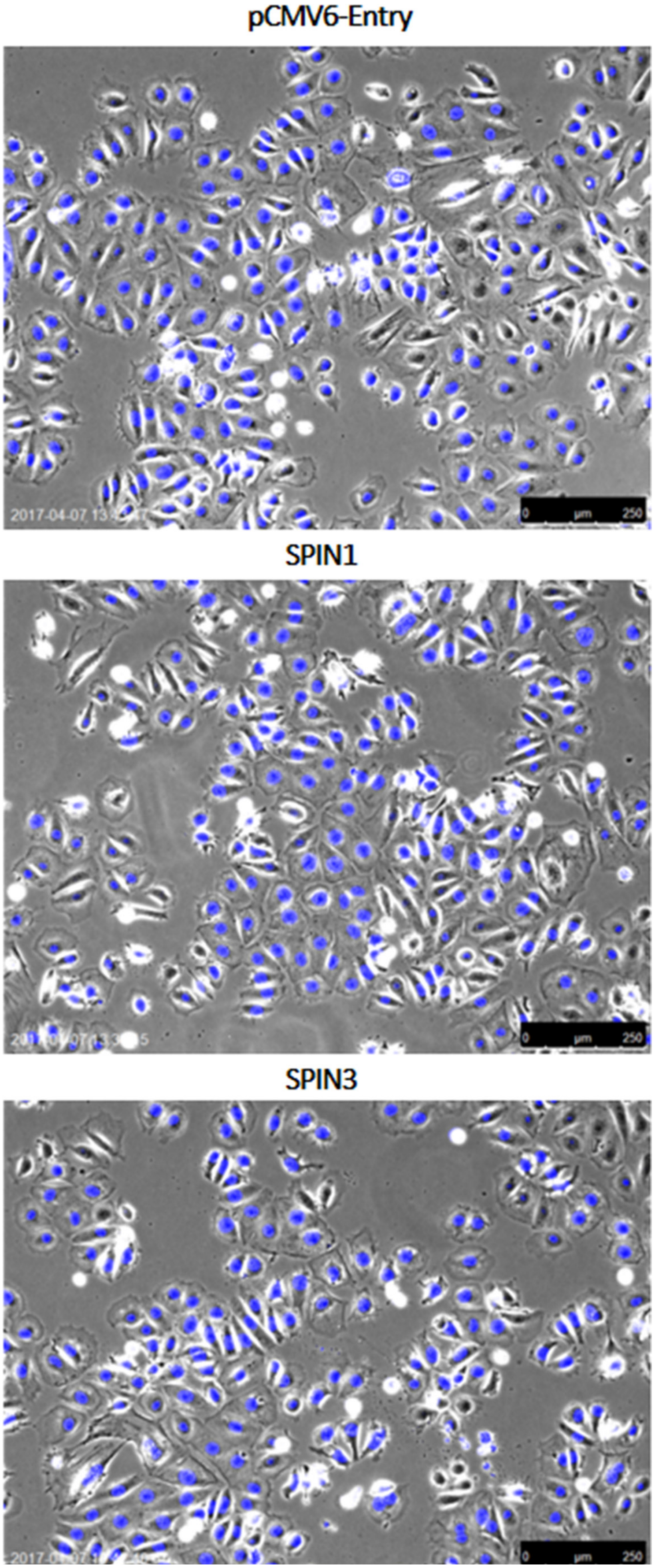

Supplementary Figure 6: TCam-2 cells do not undergo multinucleation following SPIN overexpression. Staining of cells overexpressing SPIN1 or SPIN3, or transfected with empty pCMV6-entry vector was performed using Hoechst 33258. Nuclei were visualized using a Leica DMi8 IVD microscope under UV and visible light.

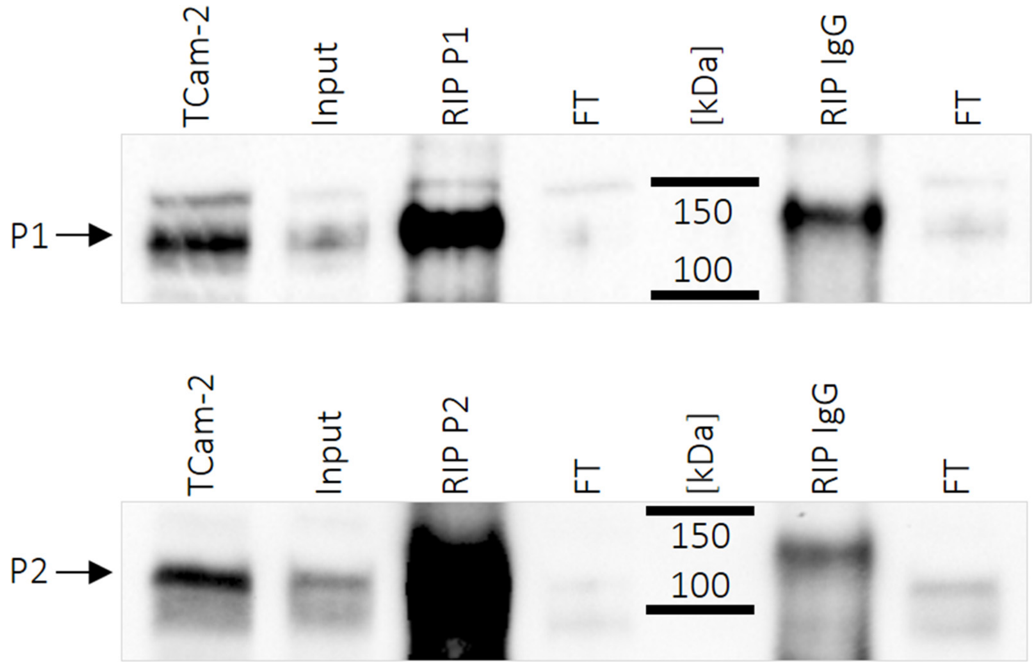

Supplementary Figure 7: Western blot detection of PUM1 and PUM2 proteins bound to beads coated with anti-PUM1 or anti-PUM2 antibodies used in the RIP experiment. Western blotting confirmed the presence of endogenous PUM1 and PUM2 proteins in inputs as well as in eluates from beads (RIP P1 and RIP P2, respectively). Their low levels were detected in flow-through (FT) as expected, while PUM proteins were absent in negative control eluate (RIP IgG).

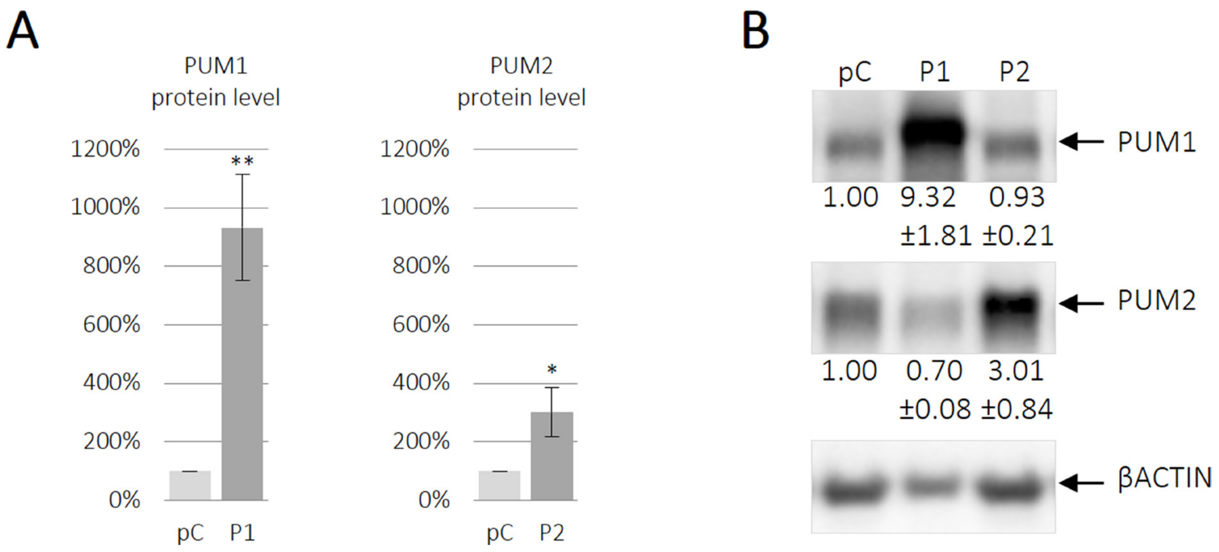

Supplementary Figure 8: Evaluation of PUM1 and PUM2 overexpression efficiencies compared to endogenous levels. PUM overexpression was analyzed in TCam-2 cells (**A**). Comparisons of endogenous and ectopic PUM levels were performed via western blotting. (**B**). $^{*}P \leq 0.05$, $^{**}P \leq 0.005$.

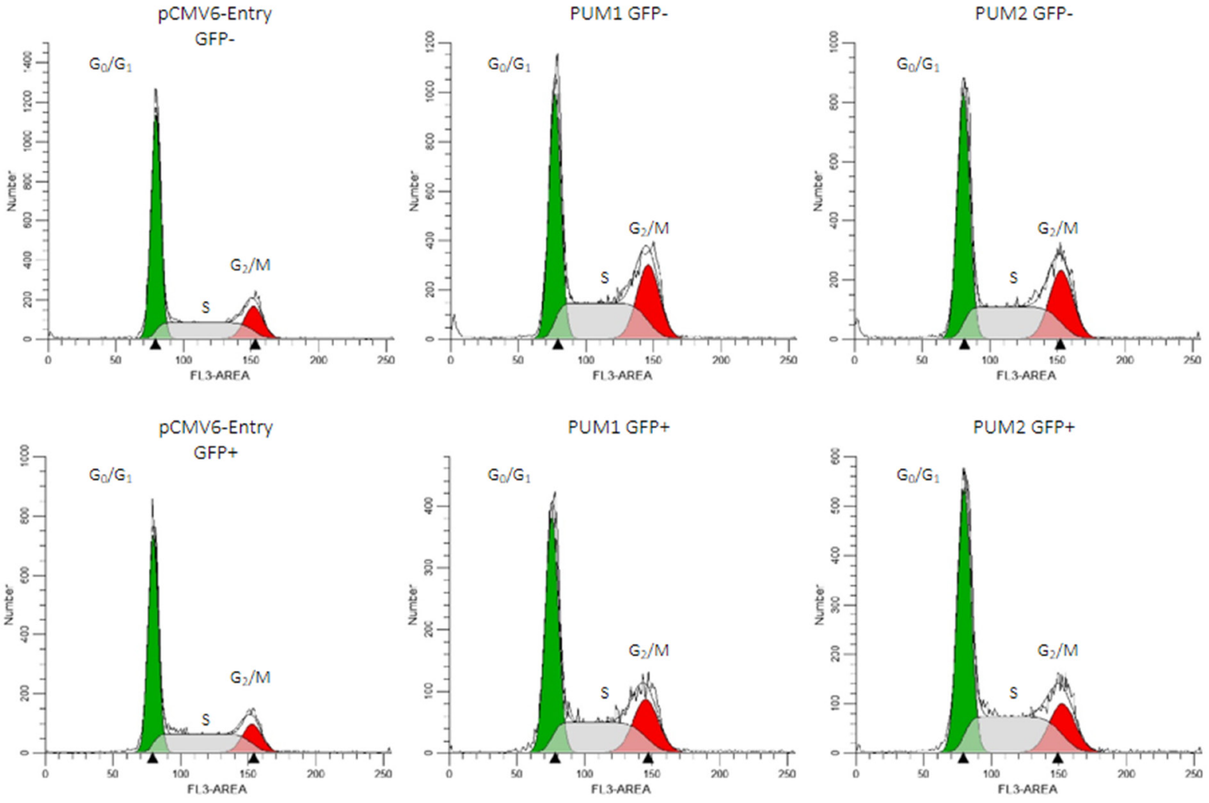

Supplementary Figure 9: Graphs showing separation quality and numbers of TCam-2 cells in different cell cycle phases as generated by ModFit LT software (Verity Software House) following PUM1 or PUM2 overexpression.
